# Tubarial salivary gland in dentistry: A review

**DOI:** 10.6026/973206300214375

**Published:** 2025-12-15

**Authors:** Shyam Sunder Madhavan Nair, Mihika Ann Menon, Sameer Chauhan, Libni D. Angel, Harshitha B, Swati Parhar, Mazen Ahmad Almasri, Rahul Tiwari, Anil Managutti

**Affiliations:** 1 Department of Dentistry, NMC Royal Hospital, Sharjah, United Arab Emirates; 2 Department of Prosthodontics Crown and Bridge, M R Ambedkar Dental College, Bangalore, Karnataka, India; 3 Department of Prosthodontics and Crown & Bridge, K. M. Shah Dental College and Hospital, Sumandeep Vidyapeeth, Vadodara, Gujarat, India; 4 Department of Pathology, Sree Balaji Medical College and Hospital, Chennai, Tamil Nadu, India; 5 Department of Periodontics, Sri Sai College of Dental Surgery, Vikarabad, Telangana, India; 6 Department of Oral Pathology, Swami Devi Dyal Hospital and Dental College, Panchkula, Haryana, India; 7 Department of Oral and Maxillofacial Surgery, Faculty of Dentistry, King Abdulaziz University, Jeddah, Saudi Arabia; 8 Department of Oral and Maxillofacial Surgery, Narsinhbhai Patel Dental College and Hospital, Sankalchand Patel University, Visnagar, Gujarat, India

**Keywords:** Tubarial salivary gland, nasopharynx, xerostomia, radiotherapy planning, dentistry

## Abstract

The tubarial salivary glands, identified in 2020 as paired mucous gland clusters located near the torus tubarius in the nasopharynx, have introduced new perspectives in salivary anatomy and oral health. Their clinical importance extends to radiology, oncology, dentistry and systemic disease involvement. Therefore, it is of interest to review current evidence on their anatomical features, imaging characteristics and clinical implications in dentistry, particularly in xerostomia, dysphagia and head-neck radiotherapy. Thus, we show that tubarial glands are relevant organs-at-risk in radiotherapy, potential sites of neoplastic change and participants in autoimmune conditions, underscoring their importance in dental practice.

## Background:

Salivary glands are vital for digestion, lubrication, antimicrobial defense and mucosal health. Traditionally, parotid, submandibular, sublingual and minor glands were recognized as principal salivary tissues. In 2020, tubarial salivary glands were described bilaterally near the torus tubarius, secreting mucus for nasopharyngeal lubrication [1, 2- 3]. Their discovery generated debate over novelty versus rediscovery, yet their macroscopic extent and clinical impact are significant [4, 5]. Radiotherapy dose to this region correlates with xerostomia, dysphagia and poor oral outcomes, establishing them as organs-at-risk [6]. Additionally, involvement in IgG4-related disease, Sjögren's syndrome and rare neoplasms highlights their systemic and dental importance [7, 8- 9]. Therefore, it is of interest to report the current evidence on the anatomy, imaging characteristics and clinical relevance of the tubarial salivary glands, with particular emphasis on their implications in dentistry.

## Results and discussion:

The tubarial glands are consistently visualized on PSMA PET/CT and histologically resemble mucous acini-dominant salivary tissue. Conventional MRI/CT provides limited visibility. These features suggest they occupy an intermediate position between major and minor glands. For dental clinicians, imaging awareness helps differentiate nasopharyngeal gland tissue from pathological lesions, supporting accurate diagnosis and care [1, 5, 6, 13, 14, 15] (Table 1 - see PDF). Radiotherapy studies demonstrate a clear dose-response relationship between tubarial gland exposure and xerostomia or dysphagia. Re-optimized IMRT successfully reduces mean dose without compromising tumor control, highlighting their role as organs-at-risk. Rare tumors have also been reported. For dentistry, these insights guide preventive strategies, caries management and long-term oral health surveillance in oncology patients [1, 2, 7, 16] (Table 2 - see PDF). Tubarial glands may act as target sites in systemic diseases. IgG4-related disease causes visible swelling that resolves with corticosteroids, while altered PSMA uptake suggests possible involvement in Sjögren's syndrome. Such findings broaden the scope of xerostomia etiologies. Dentists should consider nasopharyngeal dryness and interdisciplinary referrals when conventional salivary gland explanations are insufficient [4, 8 and 9] (Table 3 - see PDF).

## Anatomical and histological characteristics:

The emergence of the tubarial gland as a clinically significant structure has important implications for anatomy, dentistry, oncology and systemic disease management. The tubarial glands appear histologically similar to minor salivary glands, consisting of tightly packed mucous acini and sparse ducts [10]. Their location in the nasopharynx distinguishes them from other minor glands, as they form consistent bilateral clusters measurable by advanced imaging modalities [11]. Some histological studies demonstrate sex-based variations in acinar size, suggesting hormonal or genetic influences on their morphology [12]. Debate continues over whether they should be classified as new -major|| glands, as their secretions do not significantly contribute to oral saliva [13]; however, their macroscopic distinctness justifies independent clinical recognition.

## Imaging characteristics and diagnostic relevance:

PSMA PET/CT has been pivotal in identifying the tubarial glands, with consistent uptake observed in nearly all patients, establishing it as a highly reliable diagnostic marker [14]. Standard CT and MRI are less sensitive in visualizing these structures, although subtle tissue characteristics can sometimes be identified [15]. For dental professionals, awareness of these imaging features is essential for correlating xerostomia symptoms, avoiding misdiagnosis and distinguishing these glands from nasopharyngeal masses.

## Radiotherapy exposure and functional implications:

The strongest evidence for clinical relevance arises from radiotherapy studies. Higher radiation doses to the tubarial region are significantly associated with xerostomia and dysphagia 12-24 months after treatment [6]. Modified IMRT planning can reduce mean glandular dose by 5-7 Gy without compromising tumor coverage, offering a pathway to improved patient quality of life [16]. Given the oral consequences of xerostomia-such as radiation caries, candidiasis and periodontal disease-these findings hold major importance for dental teams managing oncology patients.

## Autoimmune and systemic disease associations:

Tubarial glands may also be involved in systemic autoimmune disorders. IgG4-related disease has been shown to cause visible swelling in this region, which regresses with corticosteroid therapy [7]. In addition, altered PSMA uptake patterns in patients with Sjögren's syndrome suggest possible tubarial involvement [8]. These observations broaden the diagnostic landscape for xerostomia and support multidisciplinary evaluation when conventional salivary gland pathology does not fully explain symptoms.

## Neoplastic potential and surgical considerations:

Although rare, tubarial salivary tissue can undergo neoplastic transformation. Case reports describe benign and malignant tumors arising within this nasopharyngeal cluster [9], necessitating differentiation from adenoidal or nasopharyngeal malignancies. For surgeons, anatomical awareness of these glands is essential during nasopharyngeal procedures to prevent inadvertent injury and preserve salivary function.

## Implications for dental and oral healthcare practice:

For dental practitioners, the implications are multifaceted. Recognizing tubarial gland-sparing strategies in head-and-neck radiotherapy can help anticipate patient outcomes and guide preventive protocols for radiation-related oral complications. Additionally, acknowledging tubarial gland involvement in systemic or autoimmune conditions can aid in diagnosing unexplained dryness or pharyngeal discomfort. Thus, the tubarial glands represent not merely an anatomical curiosity but a clinically relevant structure with direct consequences for oral health care.

## Conclusion:

The tubarial salivary glands extend the recognized map of salivary tissues to the nasopharynx. While their precise classification remains debated, their clinical relevance is undeniable. Radiologically, they are reliably identified with PSMA PET/CT; oncologically, they are organs-at-risk in radiotherapy whose sparing can improve xerostomia outcomes; and pathologically, they may be involved in systemic autoimmune diseases and rare tumors.
